# Sex differences in post-exercise fatigue and function in myalgic encephalomyelitis/chronic fatigue syndrome

**DOI:** 10.1038/s41598-023-32581-w

**Published:** 2023-04-03

**Authors:** Fred Friedberg, Jenna L. Adamowicz, Patricia Bruckenthal, Maria Milazzo, Sameera Ramjan, Xiaoyue Zhang, Jie Yang

**Affiliations:** 1 Department of Psychiatry, Stony Brook University, L10-060Y, 101 Nicolls Road, Stony Brook, NY 11794-8101 USA; 2 Department of Psychological and Brain Sciences, University of Iowa, 340 Iowa Avenue, G60 PBSB, Iowa City, IA 52242 USA; 3 School of Nursing, Health Sciences Center, Level 2, Stony Brook, NY 11794-8240 USA; 4 Memorial Sloan Kettering Cancer Center, 641 Lexington Avenue, 7Th Floor, New York, NY 10022 USA; 5 Renaissance School of Medicine, Stony Brook University, L3-108, 101 Nicolls Road, Stony Brook, NY 11794-8036 USA

**Keywords:** Biomarkers, Signs and symptoms

## Abstract

To assess biobehavioral sex differences in myalgic encephalomyelitis/chronic fatigue syndrome (ME/CFS) utilizing a low burden exercise protocol, 22 females and 15 males with ME/CFS and 14 healthy controls underwent two six-min walk tests. Fifteen daily assessments were scheduled for fatigue and function ratings and heart monitoring. Six-min walk tests were conducted on days 8 and 9. The ME/CFS group showed high self-report fatigue and impaired physical function, whereas healthy controls did not show fatigue or function abnormalities. In patients, no significant post-exercise changes were found for heart rate variability (HRV); however, heart rate decreased in ME/CFS males from Day 14 to Day 15 (*p* = 0.046). Female patients showed increased fatigue (*p* = 0.006) after the initial walk test, but a downward slope (*p* = 0.008) in fatigue following the second walk test. Male patients showed a decrease in self-report work limitation in the days after exercise (*p* = 0.046). The healthy control group evidenced a decrease in HRV after the walk tests from Day 9–14 (*p* = 0.038). This pilot study did not confirm hypotheses that females as compared to males would show slower exercise recovery on autonomic or self-report (e.g. fatigue) measures. A more exertion-sensitive test may be required to document prolonged post-exertional abnormalities in ME/CFS.

Trial registration: NCT NCT03331419.

## Introduction

Post-exertional malaise (PEM) which refers to prolonged symptom flare-ups after physical activity is a debilitating core symptom of myalgic encephalomyelitis/chronic fatigue syndrome (ME/CFS)^1^. PEM may lead to increased symptoms of pain and fatigue^2–6^, abnormal cardiopulmonary responses to exercise^7–9^, and negative changes in cognitive function^10,11^. PEM-related behavioral impacts in ME/CFS occur most prominently during the post-exertion recovery period. In a study of self-report PEM following a maximal exercise test^12^, 85% of healthy controls indicated full recovery within 24 h, in contrast to 0% of ME/CFS patients. Although the entire control group recovered within 2 days, 60% of CFS patients reported that it took 5 or more days to fully recover from the test. Furthermore, in an unpublished finding from a ME/CFS observational study^13^, a significant sex difference was found in patient-reported PEM, indicating that PEM was of significantly longer duration (ranging from several hours to several days) in females than males (*p* = 0.004). By comparison, overall fatigue intensity, a cardinal symptom of ME/CFS, was not significantly different between females and males.

Post-exercise cardiac autonomic abnormalities in ME/CFS have been found in a two-day repeat maximal exercise test in comparison to healthy controls^14,15^. The recovery period after the 2-day repeat exercise protocol was more likely to reveal pathophysiological changes^2–4,9^ apparently because sustained PEM, initially triggered during the first test was then exacerbated during the 2nd test. In healthy controls, full recovery occurs between test 1 and test 2^14,15^. In a recent exercise study^16^, 16 ME/CFS patients and 10 healthy controls underwent a sub-maximal warm-up followed by cardiopulmonary exercise testing on two consecutive days. A significant group effect was identified for lower post-exercise heart rate recovery (HRR) in ME/CFS patients. Furthermore, in a case control study in ME/CFS, a submaximal bicycle exercise test resulted in reduced parasympathetic reactivation during recovery, i.e. slower HRR and lower heart rate variability (HRV) in the ME/CFS group^17^.

Given the high subject burden and logistical challenges of maximal exercise tests, this pilot feasibility study utilized a modest burden six-minute walk test to potentially trigger post-exercise abnormalities in ME/CFS as evidenced by fatigue elevations and cardiac autonomic abnormalities^16,17^. Such submaximal exercise testing may be able to distinguish ME/CFS from healthy controls and expand the pool of research participants in this population to include debilitated patients who may not be able to perform a maximal exercise test^18^. Our hypothesis was that two high-effort six-minute walk tests (preceded by 30 s of fatiguing knee squats) conducted on consecutive days in ME/CFS subjects will show greater adverse impacts and slower recovery to resting baseline in females as compared to males with respect to cardiovascular autonomic functioning and symptom resolution. A healthy control group was included to compare the post-exercise trends in autonomic and behavioral measures to ME/CFS cases. Both in-person and home-based participation were utilized in this study.

## Methods

### Participant recruitment

The baseline target sample size of 40 enrolled participants was intended to achieve, after 20% expected attrition, an endpoint sample of 32 (11 males, 21 females), as 70–80% of ME/CFS patients are female^19^. The shift to remote visits did not change our recruitment targets.

Preliminary data (N = 73) from the PI’s laboratory indicated that self-report PEM scores at resting baseline (frequency x severity ratings of PEM) were positively correlated (*p* < 0.05) with fatigue ratings taken immediately after and 10 min after completion of a standard low exertion six-minute walk test. Fatigue scores at baseline increased at both 10- (*p* = 0.013) and 20-min (*p* = 0.005) after completion of the walk test. By comparison, in healthy subjects, the six-minute walk test is associated with *lower* post-walk fatigue^20^. This preliminary data suggest that the abnormal symptom exacerbations characteristic of PEM can be provoked and confirmed by patients after a brief, low effort exercise task as proposed.

Recruitment in the United States (U.S.) began in September 2017 and ended in February 2022. The study initially required in-person visits (n_1_ = 24); however, in later years, only remote visits (n_2_ = 28) were conducted. In-person subjects were recruited via local advertising, and remotely enrolled participants were reached via national advertising. Targeted participant recruitment methods included internet advertising to large patient organizations (e.g. Health Rising, Solve ME/CFS Initiative) and referrals from the CFS-specialized practice of Dr. Susan Levine which was local to the study site. This was a convenience sample.

Home-based study participation was started in 2019 due to the slow pace of recruitment and later continued with the advent of pandemic restrictions from 2020 to 2022. With home participation, the study was more likely to recruit patients who were disabled and homebound^21^. For healthy adult subjects, recruitment was done solely via internal notices posted on Stony Brook University weekly online announcements.

### Initial screening

The initial screening of prospective participants for study eligibility was conducted by the project nurses (PB, MM) utilizing a validated phone interview^22^. Selection criteria were: (a) age between 21 and 65^23,24^; (b) Fukuda-based CFS symptoms^24^ including 6 months of medically unexplained, debilitating fatigue plus 4/8 secondary symptoms, i.e., memory or concentration difficulties, unrefreshing sleep, sore throats, headache, muscle pain, joint pain, tender lymph nodes, post-exertional malaise; and (c) absence of exclusionary illnesses. To meet CFS symptom criteria, each of the 4 qualifying symptoms had to be endorsed with a frequency of “sometimes” or greater and an intensity of “moderate” or higher^25^. The phone interviewer identified medical exclusions of fatigue clearly attributable to self-report medical conditions, e.g., untreated hypothyroidism^24^. In addition, individuals were excluded if they were taking heart altering medication (e.g. beta-blockers and anti-depressants). Exclusionary psychiatric disorders included any psychosis, or alcohol/substance abuse within two years prior to illness onset and any time afterward, and current or past depression with melancholic or psychotic features within 5 years prior to onset of CFS or anytime afterward^24^.

The screening of prospective healthy participants was also conducted by the project nurses (PB, MM)^22^. These self-identified healthy individuals were required to be aged 21–65 and were excluded if they reported the presence of a chronic illness, either medical or psychiatric, and/or were taking prescription medication for an ongoing illness. All study subjects were considered physically capable of doing the exercise tasks and were willing to wear a heart monitor.

### Procedure

**Figure 1:**
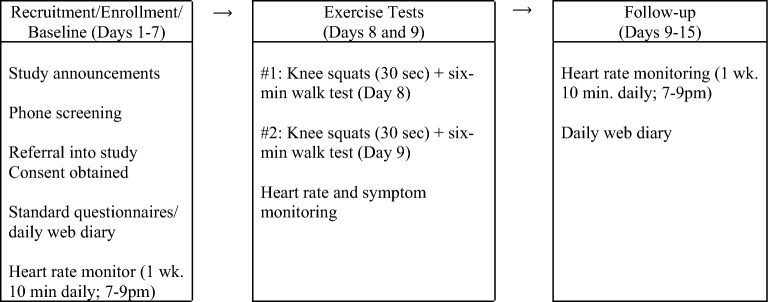
Study Timetable. This figure shows the following sequence of study activities: (**a**) recruitment, enrollment, and baseline tasks (left panel); (**b**) exercise tests of knee squats and six-min walk tests and heart rate and symptom monitoring (middle panel); and (**c**) post-exercise follow-up activities of heart rate and symptom monitoring (right panel).

The study procedures (Fig. 1) included: (a) participant eligibility phone screening followed by land mailing, completion and return of the informed consent form and standard questionnaires (pre-study baseline only); (b) mailing and return of heart monitors after data collection was complete; (c) 15 days of online fatigue and functional limitation ratings and home-based HRV monitoring (10 min/day); (d) Staff-guided instructions (in person or via phone call) using a written stepwise protocol to guide each participant through study procedures.

Instructions to participants included attachment of the heart monitor^13^, use of the online web diary, and conduct of the knee squats and the two walk tests. Participants were asked to abstain from caffeine and vigorous exercise for 24 h and from eating for 3 h prior to the walk tests. These instructions with demonstration video on how to do self-paced knee squats were given:

Stand with your feet slightly wider than your hips. Toes pointed slightly outward. Your weight should be on your heels and the balls of the feet, as if you were pasted to the ground (as if about to sit down). Now look straight ahead and pick a spot on the wall in front of you. Look at this spot the entire time you squat, not looking down at the floor or up at the ceiling. As you squat, keep your upper body straight. Don’t bend forward. Ready. Go ahead.

The 30 s of knee squats, intended to increase exertional impacts, was followed by a maximum effort (“*walk as fast as you can*”) six-min walk test scheduled for days 8 and 9. The home-based tape-measured walking course consisted of repeated laps on relatively straight paths, i.e. zig zags allowed. The straightaway section varied in length depending on house configuration. These repeat physical exercise tasks were intended to trigger PEM, given that post-exertion impacts can result from relatively minor physical effort in ME/CFS individuals^18^. This study was approved by the Stony Brook University Committee on Research Involving Human Subjects and all participants provided informed consent. All methods were performed in accordance with the relevant guidelines and regulations.

### Standard questionnaires

#### Fatigue severity scale (FSS)

This measure of the effect of fatigue on functioning is comprised of nine items rated on a seven-point Likert-type rating scale, where one indicates no impairment and seven indicates severe impairment (score range: 1.00–7.00). In the initial validation study^26^, internal consistency for the FSS was excellent (Cronbach’s α = 0.80) and the scale clearly distinguished between patients and controls. The scale, recommended for use in CFS^27^, showed high alpha consistency in our sample (Cronbach’s α = 0.87).

#### SF-36 physical function subscale (PFS)

The PFS^28^ of the SF-36 measures physical limitations of ill health on a scale of 0 to 100, where 0 indicates limited in all activities, including basic self-care and 100 indicates no limitations. The PFS is composed of ten items where each item is scored on the basis of the limitations perceived by surveyed individuals. Item scores (1, 2, or 3) are summed to obtain a total score. Normative values are available for the PFS for the U.S. population. This subscale has shown good internal consistency (Cronbach’s α ≥ 0.81) in psychometric studies^28^ and in our sample (α = 0.88).

### Online web diary (Day 1 to Day 15)

The online web diary (StudyTrax, Inc., Macon, Georgia) was scheduled daily and directed participants to rate fatigue intensity and 3 types of activity limitations for job and home activities at the end of each study day (work, housekeeping, exercise). Both fatigue intensity and activity limitations were ordinal variables with integer values from 0 (None/No limitation) to 10 (Highest/Major limitation).

### Objective measures

#### Knee squats

Knees squats are a type of resistance training that have been shown to increase perceptions of both effort and fatigue e.g.^29^, in healthy populations. Thirty seconds of knee squats were scheduled immediately before each six-minute walk test in order to increase post-exertion fatigue and potential autonomic dysfunction triggered by the walk tests.

#### Six-minute walk test

This is a sub-maximal exercise test of functional capacity^30,31^ which measures the distance walked during a six-minute interval. The test is a useful and reproducible measure of exercise tolerance which does not require expensive apparatus^31^. To enhance post-exertional impacts, subjects were instructed to *“walk as fast as you can*.”

#### Heart rate monitor (Day 1 to Day 15)

Heart rate variability (HRV) and heart rate (HR) are non-invasive measures of cardiac autonomic function. Both variables were calculated via electrocardiogram (ECG) collected with a research-grade multifunction ambulatory heart monitor (eMotion Faros 180; MegaElectronics, Kuopio, Finland). ECG data were collected at a 500 Hz sampling rate using a 3-lead configuration. Data were exported for analysis in the Kubios HRV analysis suite (version 3) for R-peak detection and the visual inspection of artifacts (e.g. non-sinus beats, movement), and HRV calculation. R-peaks were detected using a modified Pan-Tompkins algorithm^32,33^. Detected artifacts were corrected by replacing artifacts with interpolated values via cubic spline interpolation.

The root mean square of successive differences (RMSSD) was then calculated to estimate HRV. RMSSD reflects beat-to-beat variance in heart rate and is the primary time-domain measure used to estimate the vagally mediated changes reflected in HRV^34^*.* To approximate a standard setting, HR data were collected daily by participants at home while sitting in a comfortable chair for ten minutes in the evening between 7 pm and 9 pm. Subject were instructed to be in “quiet time” (no other activity, including TV) during resting HRV assessment.

This small beeper-size heart monitor was sent to each participant. Proper electrode placement was guided by written and pictorial instructions and confirmed with a staff phone call to each participant. Consistent with recommendations^35^, inter-beat intervals recorded for five min (or more) are sufficient to approximate parasympathetic outflow to the heart^36^. The first two minutes of data were discarded to provide an acclimatization period.

### Data analysis

Daily data for all measures were collected from Day 1 to Day 15 for the HR autonomic measure, fatigue ratings (0–10), and ratings (0–10) of functional limitations regarding work, housekeeping and exercise limitations. Day 1 to Day 8 were baseline days and Day 9 to Day 15 were walk test and post-walk test days. The “baseline” value was the average for each variable from Day 1 to Day 8. Analyses focused on aggregate baseline data (days 1–8) and the data for each day from Day 9 to Day 15. All outcome variables were treated as continuous variables in order to analyze linear trends over time.

Piecewise linear mixed effect models^37^ were used to analyze the daily longitudinal data (HRV, HR, fatigue intensity, three types of activity limitations). To compare the differences in daily measurements between female and males in the ME/CFS group, fixed effects adjusted in the models were for gender, a time variable, a time spline variable, an interaction term between gender and time, and an interaction term between gender and time spine variable. Time was a continuous variable with integer value 0 to 7 to represent the baseline (0) and time points from Day 9 (1) to Day 15 (7). Time spline was also a continuous variable with value 0 to 7 to divide time into 2 segments. The breakpoint in the linear trend was decided by using the smallest model goodness-of-fit statistics^38^. Similarly, piecewise linear mixed effect models with adjustment for treatment group were utilized to compare daily data between ME/CFS and health control groups. Based on Akaike Information Criteria (AIC), the covariance structure to model correlation among longitudinal measurements from the same patient is selected from Compound Symmetry (CS), and first-order autoregressive (AR(1)), Toeplitz (TOEP), and Unstructured (UN). Statistical analysis was performed using SAS 9.4 (SAS Institute Inc., Cary, NC) and significance level was set at 0.05.

## Results

### Participant characteristics

Of the 118 phone-screened individuals, 67 (56.8%) were excluded for not meeting entry criteria as follows: subthreshold CFS symptoms (33/67), self-report exclusionary medical/psychiatric illness (22/67), and other factors, e.g. age or BMI out of range (12/67). Fifty-one individuals were enrolled and 9 (17.3%) were lost to follow-up. Most participants (Table 1) were in their 40 s, white (90.3%), and female. The majority of ME/CFS participants were ill for over a decade. Fourteen subjects were healthy controls. For the ME/CFS group, standard questionnaires showed high fatigue severity and impaired physical function. Healthy controls did not show fatigue or function abnormalities.

**Table 1:** Demographic information of study participants.

	ME/CFS		Healthy controls	
	Females	Males	Females	Males
N	22	15	141	3
Age	45.8 (11.6)	51.6 (12.1)	46.7 (13.9)	25 (5)
Illness duration	15.8 (9.6)	15.1 (11.7)	–	–
FSS	6.7 (0.41)	6.7 (0.33)	1.78 (0.385)	2.06 (0.97)
SF-36 PF	57.9 (16.5)	55.5 (12.6)	96.3 (2.7)	100 (0)

Illness duration is in years.

*FSS* fatigue severity scale, *SF-36 PF* short form 36, physical functioning subscale.

Numbers in parentheses are standard deviations.

Symptom presentations of ME/CFS participants were consistent with the findings of a large sample factor analytic study of diagnostic criteria in ME/CFS^39^. This study identified these apparent core symptom dimensions: cognitive dysfunction and post-exertional malaise (each endorsed by 90 + %) and sleep dysfunction (79%). Our screening data also showed high levels of endorsement for impaired memory or concentration (93%; N = 40), post-exertional malaise (100%; N = 43) and unrefreshing sleep (97.7%; N = 42). Compliance for 15 days of web diary data collection was 92.1% for female patients, 84.9% for male patients, and 86.7% for healthy controls. Six-min walk test distances for ME/CFS participants averaged over 2 walk tests for males was 342.62 m (SD: 129.12) and for females, 382.28 m (SD: 120.77). Walk test distances were not recorded for healthy controls. For knee squats in the ME/CFS group, the number of squats averaged over two 30 s sessions was 11.25 (SD: 3.58) for males and 10.86 (SD: 2.79) for females.

### Sex differences within the ME/CFS group

**Figure 2:**
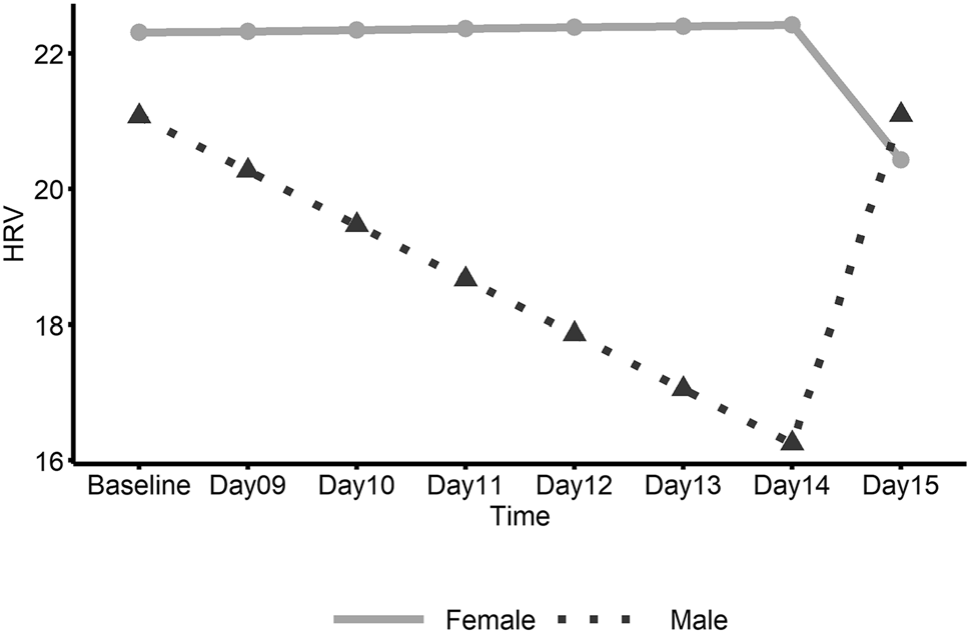
Linear trend of heart rate variability (HRV) by gender within the ME/CFS group. This graph shows daily heart rate variability (HRV) in ME/CFS participants from pre-exercise Baseline (Days 1–8) to follow-up (Days 9–15) with the solid line representing females and the dashed line representing males.

**Figure 3:**
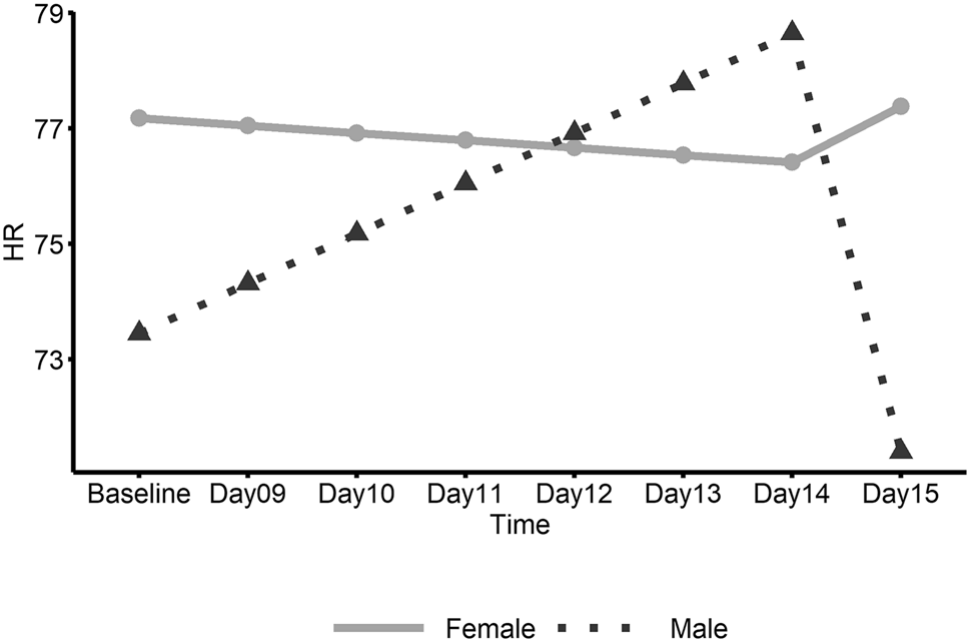
Linear trend of heart rate (HR) by gender within the ME/CFS group. This graph shows daily heart rate (HR) in ME/CFS participants from pre-exercise Baseline (Days 1–8) to follow-up (Days 9–15) with the solid line representing females and the dashed line representing males.

**Figure 4:**
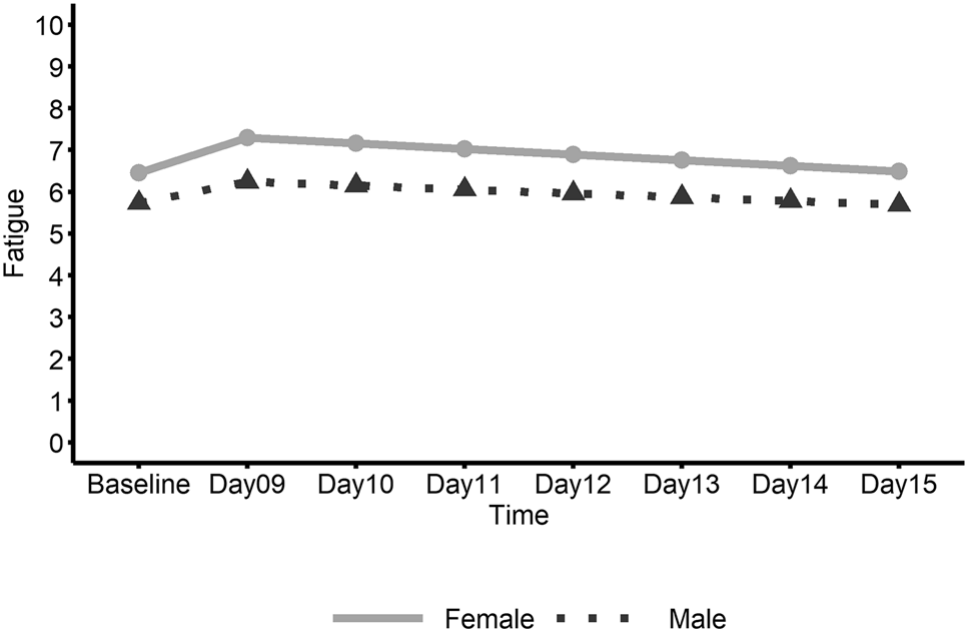
Linear trend of fatigue ratings by gender in the ME/CFS group. This graph shows daily fatigue ratings in ME/CFS participants from pre-exercise Baseline (Days 1–8) to follow-up (Days 9–15) with the solid line representing females and the dashed line representing males.

Female and male data in the ME/CFS group for pre-walk test baseline and post-walk test days 9–15 are shown in Table 2. Figures 2, 3 and 4 show the estimated linear trends by sex for HRV, HR and fatigue, respectively, based on piecewise linear mixed effect models.

**Table 2:** Average heart parameter values and self-report ratings for female and male ME/CFS patients.

Gender	Visit	HRV/RMSSD*	Heart rate	Fatigue^+^	Work limitation^+^	Housekeep limitation^+^	Exercise limitation^+^
Female	Baseline^#^	25.558	75.936	6.5	6.4	6.4	7.2
	Day 09^1^	27.938	76.787	7.6	7.0	6.9	7.2
	Day 10^2^	26.836	75.747	7.2	6.6	6.9	7.6
	Day 11	24.082	76.643	6.8	6.2	6.4	7.2
	Day 12	24.237	74.467	6.7	6.1	6.2	7.5
	Day 13	23.547	80.780	7.1	6.8	6.9	8.0
	Day 14	23.546	74.914	6.8	6.7	6.2	7.5
	Day 15	22.672	78.839	6.5	6.3	6.4	7.5
	Mean	24.94	76.64	6.89	6.50	6.54	7.45
	(SD)	(16.49)	(13.30)	(1.86)	(2.72)	(2.52)	(2.27)
Male	Baseline^#^	21.071	73.372	5.7	5.8	4.7	7.0
	Day 09^1^	18.065	74.764	6.2	5.2	4.5	7.0
	Day 10^2^	21.220	74.549	6.7	6.8	5.5	7.4
	Day 11	19.240	74.472	6.1	6.4	5.3	7.6
	Day 12	15.716	82.502	5.6	5.7	5.1	7.2
	Day 13	17.412	75.677	5.4	5.3	4.5	7.1
	Day 14	18.254	75.025	5.9	5.5	4.7	7.1
	Day 15	22.746	69.651	5.7	5.8	4.9	7.5
	Mean	19.20	75.10	5.91	5.80	4.88	7.21
	(SD)	(8.47)	(13.26)	(2.14)	(3.09)	(2.54)	(2.56)

*Heart rate variability/root mean square of the standard deviation.

^+^Ratings (0–10).

^#^Baseline is the average value from Day 1 to Day 8 (pre-walk test).

^1^First six-minute walk test.

^2^Second six-minute walk test; Standard deviations are in parentheses.

No significant changes over time were found for HRV (Table 3); however, heart rate significantly decreased in ME/CFS males from Day 14 to Day 15 (Fig. 3) and the slope change in heart rate at Day 14 was also significant. Female patients showed a significant increase in average fatigue rating from baseline to the first day (Day 9) after baseline which included the initial walk test (Fig. 4). Also, for ME/CFS females, a significant negative slope for fatigue was found from Day 9 to Day 15. In addition, the change in slope for fatigue at Day 9 was significant. No other significant linear trends were found for females. Male patients did not show significant linear trends for fatigue but did show a significant decrease (E = − 0.184; CI − 0.37– − 0.00; *p* = 0.046) in self-report work limitation from the 2nd day after baseline, i.e. Day 10 to Day 15.

**Table 3:** Estimated parameters of linear trend from baseline to Day 15 between genders within ME/CFS group.

Gender	Parameter	Estimates	95% CI	*P*-value
Heart rate variability (HRV)				
Female	Intercept	22.309	17.07–27.55	
	Slope [Before Day 14]	0.019	− 0.59–0.63	0.9495
	Slope [After Day 14]	− 1.991	− 10.50–6.52	0.6291
	Slope change [at Day 14]	− 2.010	− 10.48–6.46	0.6243
Male	Intercept	21.075	13.60–28.55	
	Slope [Before Day 14]	− 0.804	− 1.67–0.06	0.0669
	Slope [After Day 14]	4.840	− 7.65–17.33	0.4290
	Slope change [at Day 14]	5.644	− 6.85–18.13	0.3578
Female vs. Male	Slope [Before Breakpoint]	0.823	− 0.23–1.88	0.1214
	Slope [After Breakpoint]	− 6.831	− 21.94–8.28	0.3568
	Slope change [at Breakpoint]	− 7.654	− 22.74–7.43	0.3025
Heart rate (HR)				
Female	Intercept	77.177	71.69–82.67	
	Slope [Before Day 14]	− 0.128	− 0.84–0.58	0.7223
	Slope [After Day 14]	0.971	− 3.93–5.88	0.6965
	Slope change [at Day 14]	1.098	− 4.15–6.35	0.6801
Male	Intercept	73.444	65.54–81.34	
	Slope [Before Day 14]	0.868	− 0.15–1.89	0.0948
	Slope [After Day 14]	− 7.255	− 14.39–− 0.12	**0.0463**
	Slope change [at Day 14]	− 8.123	− 15.79–− 0.45	**0.0380**
Female vs. Male	Slope [Before Breakpoint]	− 0.995	− 2.24–0.25	0.1153
	Slope [After Breakpoint]	8.226	− 0.43–16.88	0.0625
	Slope change [at Breakpoint]	9.221	− 0.07–18.51	0.0518
Fatigue				
Female	Intercept	6.455	5.62–7.29	
	Slope [Before Day 9]	0.841	0.24–1.45	**0.0065**
	Slope [After Day 9]	− 0.134	− 0.23–− 0.04	**0.0080**
	Slope change [at Day 9]	− 0.976	− 1.63–− 0.32	**0.0038**
Male	Intercept	5.733	4.72–6.75	
	Slope [Before Day 9]	0.508	− 0.23–1.25	0.1789
	Slope [After Day 9]	− 0.092	− 0.22–0.03	0.1446
	Slope change [at Day 9]	− 0.600	− 1.41–0.21	0.1465
Female vs. Male	Slope [Before Breakpoint]	0.333	− 0.62–1.29	0.4939
	Slope [After Breakpoint]	− 0.043	− 0.20–0.12	0.5977
	Slope change [at Breakpoint]	− 0.375	− 1.42–0.67	0.4795

Significant values are in bold.

### Trends in the ME/CFS treatment group and healthy control group

**Figure 5:**
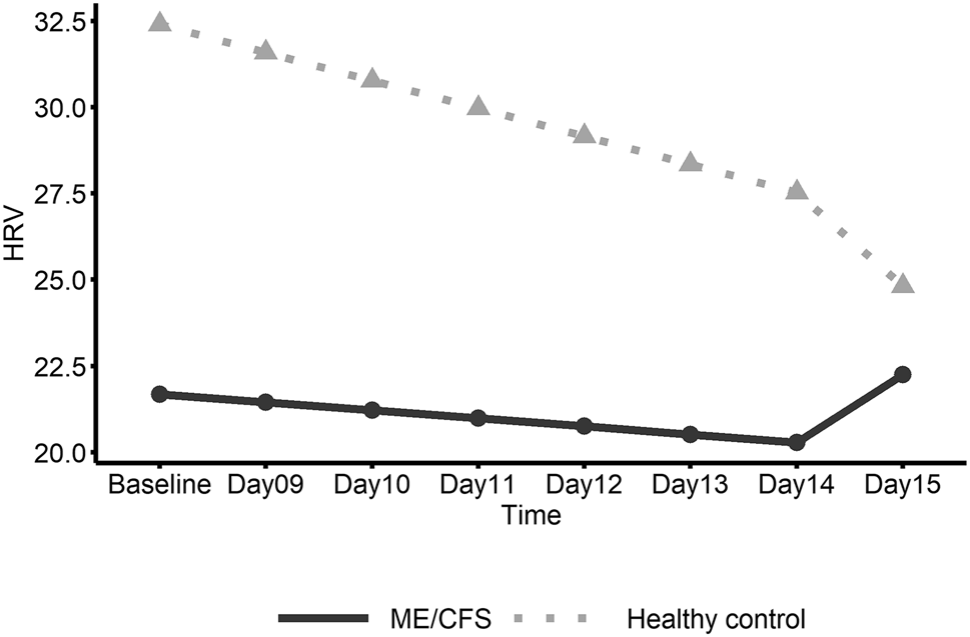
Linear trend of heart rate variability (HRV) for ME/CFS and healthy control groups. This graph shows daily heart rate variability (HRV) in ME/CFS and healthy control participants from pre-exercise Baseline (Days 1–8) to follow-up (Days 9–15) with the solid line representing the ME/CFS group and the dashed line representing healthy controls.

**Figure 6:**
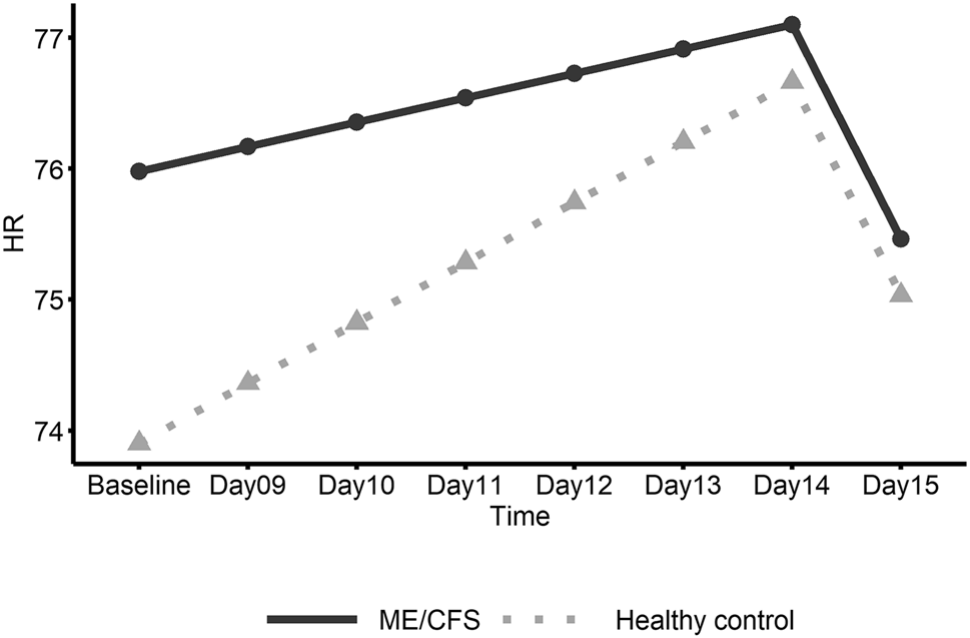
Linear trend of heart rate (HR) for ME/CFS and healthy control groups. This graph shows daily heart rate (HR) in ME/CFS participants from pre-exercise Baseline (Days 1–8) to follow-up (Days 9–15) with the solid line representing the ME/CFS group and the dashed line representing healthy controls.

**Figure 7:**
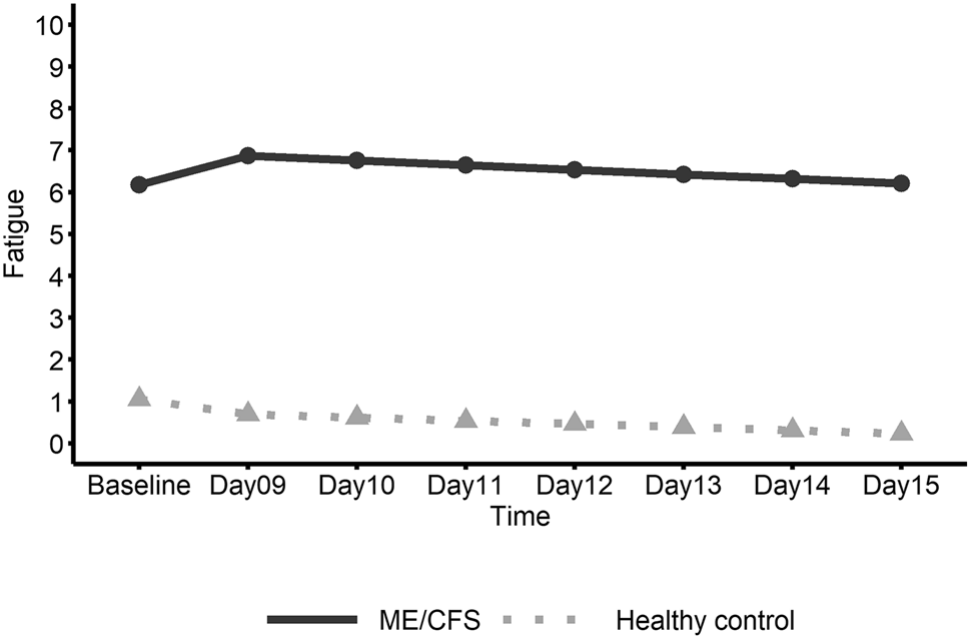
Linear trend of fatigue for ME/CFS and healthy control groups. This graph shows daily fatigue ratings in ME/CFS and healthy control groups from pre-exercise Baseline (Days 1–8) to follow-up (Days 9–15) with the solid line representing the ME/CFS group and the dashed line representing healthy controls.

Average parameter values for heart variables and self-report fatigue and functional limitations for ME/CFS and healthy control groups are listed in Table 4. Regression analysis results based on piecewise linear mixed effect models are presented Table 5. Figures 5, 6 and 7 show the plots of the estimated linear trends of HRV, heart rate, and fatigue, respectively, from baseline to post-walk test days 9 to 15 for the ME/CFS and healthy control groups.

**Table 4:** Average heart parameter values and self-report fatigue and functional limitation ratings (0–10) from baseline to Day 15 for ME/CFS and healthy control groups.

Group	Visit	HRV/RMSSD*	Heart rate	Fatigue^+^	Work limitation^+^	Housekeep limitation^+^	Exercise limitation^+^
ME/CFS	Baseline^#^	24.062	75.081	6.2	6.1	5.7	7.1
	Day 09^1^	24.534	76.089	7.1	6.3	5.9	7.1
	Day 10^2^	24.964	75.348	7.0	6.7	6.4	7.5
	Day 11	22.353	75.868	6.5	6.2	5.9	7.3
	Day 12	21.287	77.249	6.2	5.9	5.7	7.4
	Day 13	21.595	79.156	6.4	6.2	6.0	7.6
	Day 14	22.002	74.947	6.4	6.2	5.5	7.3
	Day 15	22.696	75.777	6.2	6.1	5.8	7.5
	Mean	23.03	76.2	6.50	6.22	5.87	7.35
	(SD)	(14.56)	(13.28)	(2.03)	(2.89)	(2.65)	(2.39)
Healthy control	Baseline^#^	30.663	75.490	1.0	0.1	0.1	0.5
	Day 09^1^	30.275	71.369	0.6	0.0	0.0	0.4
	Day 10^2^	21.660	75.219	0.7	0.2	0.0	0.4
	Day 11	26.326	74.595	0.7	0.0	0.0	0.4
	Day 12	26.774	75.248	0.3	0.0	0.0	0.2
	Day 13	27.252	74.535	0.2	0.2	0.0	0.2
	Day 14	25.960	78.354	0.5	0.0	0.3	0.6
	Day 15	24.603	74.409	0.2	0.0	0.0	0.5
	Mean	26.82	74.89	0.54	0.07	0.04	0.39
	(SD)	(12.83)	(10.67)	(1.11)	(0.38)	(0.31)	(1.16)

*Heart rate variability/root mean square of the standard deviation.

^+^Ratings (0–10).

^#^Baseline is the average value from Day 1 to Day 8 (pre-walk test).

^1^First six-minute walk test.

^2^Second six-minute walk test; Standard deviations are in parentheses.

**Table 5:** Estimated parameters in analysis of trend from baseline to Day 15 between ME/CFS and healthy control group.

Group	Parameter	Estimates	95% CI	*P*-value
Heart rate variability (HRV)				
ME/CFS	Intercept	21.688	17.11–26.27	
	Slope [Before Day 9]	− 0.234	− 0.76–0.29	0.3705
	Slope [After Day 9]	1.965	− 3.49–7.42	0.4675
	Slope change [at Day 9]	2.199	− 3.35–7.74	0.4249
Healthy control	Intercept	32.406	25.61–39.20	
	Slope [Before Day 9]	− 0.813	− 1.58–− 0.05	**0.0379**
	Slope [After Day 9]	− 2.721	− 10.48–5.04	0.4784
	Slope change [at Day 9]	− 1.908	− 9.79–5.98	0.6245
ME/CFS vs. Healthy control	Slope [Before Breakpoint]	0.579	− 0.35–1.51	0.2132
	Slope [After Breakpoint]	4.686	− 4.80–14.17	0.3203
	Slope change [at Breakpoint]	4.107	− 5.53–13.74	0.3911
Heart rate (HR)				
ME/CFS	Intercept	75.980	71.81–80.15	
	Slope [Before Day 9]	0.187	− 0.35–0.73	0.4954
	Slope [After Day 9]	− 1.640	− 5.39–2.11	0.3893
	Slope change [at Day 9]	− 1.827	− 5.84–2.19	0.3709
Healthy control	Intercept	73.903	67.69–80.12	
	Slope [Before Day 9]	0.460	− 0.33–1.25	0.2531
	Slope [After Day 9]	− 1.633	− 7.05–3.78	0.5532
	Slope change [at Day 9]	− 2.094	− 7.91–3.72	0.4788
ME/CFS vs. Healthy control	Slope [Before Breakpoint]	− 0.273	− 1.23–0.68	0.5745
	Slope [After Breakpoint]	− 0.007	− 6.59–6.58	0.9983
	Slope change [at Breakpoint]	0.266	− 6.80–7.33	0.9409
Fatigue				
ME/CFS	Intercept	6.180	5.59–6.77	
	Slope [Before Day 9]	0.690	0.25–1.13	**0.0023**
	Slope [After Day 9]	− 0.110	− 0.20–− 0.02	**0.0213**
	Slope change [at Day 9]	− 0.800	− 1.28–− 0.32	**0.0012**
Health control	Intercept	1.053	0.09–2.02	
	Slope [Before Day 9]	− 0.357	− 1.07–0.36	0.3257
	Slope [After Day 9]	− 0.078	− 0.23–0.07	0.3010
	Slope change [at Day 9]	0.279	− 0.50–1.05	0.4781
ME/CFS vs. Healthy control	Slope [Before Breakpoint]	1.046	0.21–1.88	**0.0147**
	Slope [After Breakpoint]	− 0.033	− 0.21–0.14	0.7130
	Slope change [at Breakpoint]	− 1.079	− 1.99–− 0.17	**0.0205**

Significant values are in bold.

Based on the analysis of the ME/CFS and healthy control groups (Table 5), HRV (Fig. 5) showed no significant changes in the ME/CFS group; however, the healthy control group showed a significant decrease in HRV after the walk tests from Day 9 to Day 14. No significant changes were found for heart rate. Regarding fatigue (Fig. 7), the ME/CFS group first showed a significant increasing pattern from baseline to Day 9 and then a significant decreasing pattern from post walk test Day 9 to Day 15. The fatigue slope change in the ME/CFS group (Fig. 7), which occurred on the 1st day after the initial walk test (Day 9) was also significant.

The ME/CFS and healthy control groups showed significantly different trend slopes for fatigue from baseline to Day 9. Also, these two groups showed different patterns of slope change at Day 9. Regarding self-report exercise limitation, ME/CFS patients showed a significant change in trend slope at post-baseline (Day 13) (E = − 0.281; CI − 0.53–− 0.03; *p* = 0.029). The ME/CFS and healthy control groups showed different trend slopes for exercise limitation after the Day 13 breakpoint (E = − 0.327; CI − 0.61–− 0.05; *p* = 0.023). In addition, these two groups showed differential changes in slope for exercise limitation ratings at the breakpoint Day 13 (E = − 0.524; CI − 0.94–− 0.11; *p* = 0.015).

## Discussion

This pilot study utilized the low burden six-minute walk test in order to identify potential post-exercise abnormalities in ME/CFS with a focus on biobehavioral sex differences. Our hypotheses that females as compared to males would show slower recovery to baseline on autonomic (HRV and heart rate) or self-report (e.g. fatigue) measures was not confirmed, although females showed a significant short-term linear trend of higher post-exercise fatigue, whereas no post-exercise fatigue trends were found in male ME/CFS participants. Heart rate significantly decreased in male ME/CFS participants on the last 2 days of post-exercise tracking. The aggregate ME/CFS patient group showed significantly increasing fatigue from baseline to the first walk test day followed by significantly decreasing fatigue to baseline levels by seven days post-exercise. The healthy control group evidenced a significant decrease in HRV in the seven days after the walk tests, whereas the ME/CFS group showed no significant HRV change. The ME/CFS and healthy control groups showed significantly different trend slopes for fatigue (up for ME/CFS; down for healthy controls) from baseline to the first walk test day.

A systematic review of cardiac autonomic dysfunction in ME/CFS reported on significant differences in HRV and other heart parameters between patients and healthy controls in both exercise and non-exercise designs^40^. By comparison, our modest post-exercise findings indicate that more effortful exercise tests may be necessary, such as maximal tests or longer duration and/or higher intensity submaximal tests, to produce enduring symptoms and cardio-autonomic abnormalities. The minimal level of physical exercise required to sustain PEM and potential biological abnormalities over multiple days is currently a matter of speculation. Perhaps the established and easy to administer 12-min walk test^41^ with instructions to “walk as fast as you can” would be a more effortful and perhaps more productive alternative to the six-minute walk test. Developing a home-based walk test protocol for PEM would potentially open up research access to many disabled ME/CFS patients who lack the mobility to participate in laboratory-based studies.

Apart from identifying a useful minimal exercise test, the issue of sex differences in exercise requires comment. In healthy individuals, a study by Mendonca et al.^42^ demonstrated that cardiac autonomic function of women is more affected by brief intense exercise than that of men. Even though both sexes showed a significant modification in HRV five min post-exercise, women showed a greater change in the HRV Low Frequency/High Frequency ratio than men from rest to recovery. Furthermore, sex differences in healthy subjects during exercise recovery have been found following a moderate effort 3 min cycling task at 60% maximum heart rate^43^. Specifically, the mean arterial pressure decreased more in females than males during the first five minutes after exercise. Perhaps biological sex differences in response to intense but brief exercise may be too short-lived to be captured in multi-day assessments. In addition, the data published here do not support use of HRV in studies trying to understand longer duration exercise intolerance or PEM.

In ME/CFS, sex differences in daily fatigue ratings following short walk tests may be due to differences in disease and/or differences in how females experience and present symptoms in comparison to males^44^. Biological studies of prolonged post-exercise sex differences in ME/CFS have not been published as far as we are aware. A report on multi-day post-exercise symptoms in a relatively small sample^45^ of ME/CFS and healthy controls utilizing a single maximal exercise test^45^ found no symptom increases in the ME/CFS group until day 5 post-exercise. Computerized watches were utilized to track daily fatigue. By comparison, days 5–7 post-exercise in the current study evidenced declining trends in fatigue and heart rate and increased HRV. These post-exertional differences in fatigue and cardio-autonomic status between these two studies may reflect the use of very different provocation tests.

Finally, the significant downtrend in post-walk test HRV for healthy controls was not expected. Conceivably this may reflect a less than robust overall health status (e.g. unhealthy diet is associated with lower HRV)^46^ in the healthy controls, given that their HRV values (20–30 ms RMSSD) were well below short-term measurement norms^47,48^ that are largely based on individuals over 40 years of age, similar to the current study. However, physical deconditioning could not be compared between patients and healthy controls given that walk test distances were not recorded for control subjects. Thus, differences between the two groups could be in part attributable to deconditioning in addition to illness factors.

The ME/CFS HRV values (RMSSD) in this study are similar to those published in our previous prospective study^13^ which collected weekly HRV data over 6 months from primarily female subjects with ME/CFS. However, the relatively small sample size in this study yielded less than definitive findings, particularly for possible sex differences. Although our home-based walk tests have shown feasibility, a more exertion-sensitive test may be needed for remote studies that seek to produce post-exertional malaise and potential biological correlates. This is particularly important for disabled, home bound patients with ME/CFS who are under-represented in scientific studies.

This pilot study utilizing a modest burden six-minute walk test to provoke prolonged post-exertional fatigue and autonomic abnormalities did not confirm hypotheses that females as compared to males would show slower recovery to baseline on autonomic (HRV and heart rate) or self-report (e.g. fatigue) measures, although females showed a significant short-term increasing trend of higher post-exercise fatigue. The aggregate ME/CFS group showed increasing fatigue from baseline to the first walk test day followed by decreasing fatigue to baseline levels by day 15. The ME/CFS and healthy control groups showed opposite trends in fatigue (up for ME/CFS; down for healthy controls) from baseline to the day of the first walk test. Clearly a more exertion-sensitive test may be required to document prolonged post-exertional fatigue and autonomic abnormalities in ME/CFS.

## Data Availability

The datasets used and/or analyzed during the current study are available from the corresponding author on reasonable request.

## Abbreviations

AIC: Akaike information criteria
AR(1): First-order autoregressive
CS: Compound symmetry
ECG: Electrocardiogram
FSS: Fatigue severity scale
HR: Heart rate
HRV: Heart rate variability
ME/CFS: Myalgic encephalomyelitis/chronic fatigue syndrome
PEM: Post-exertional malaise
PFS: SF-36 physical function subscale
RMSSD: Root mean square of successive differences
TOEP: Toeplitz
UN: Unstructured
U.S.: United States
